# On the Selectivity of Simultaneous CO_2_ and N_2_ Reduction Using TiO_2_/Carbon Sphere Photocatalysts Prepared by Microwave Treatment and Mounted on Silica Cloth

**DOI:** 10.3390/ma16175810

**Published:** 2023-08-24

**Authors:** Ewelina Kusiak-Nejman, Katarzyna Ćmielewska, Iwona Pełech, Ewa Ekiert, Piotr Staciwa, Daniel Sibera, Agnieszka Wanag, Joanna Kapica-Kozar, Marcin Gano, Urszula Narkiewicz, Antoni W. Morawski

**Affiliations:** 1 Department of Inorganic Chemical Technology and Environment Engineering, Faculty of Chemical Technology and Engineering, West Pomeranian University of Technology in Szczecin, Pułaskiego 10, 70-322 Szczecin, Poland; iwona.pelech@zut.edu.pl (I.P.); ewa.dabrowa@zut.edu.pl (E.E.); piotr.staciwa@zut.edu.pl (P.S.); agnieszka.wanag@zut.edu.pl (A.W.); joanna.kapica@zut.edu.pl (J.K.-K.); urszula.narkiewicz@zut.edu.pl (U.N.); antoni.morawski@zut.edu.pl (A.W.M.); 2 Department of General Civil Engineering, Faculty of Civil and Environmental Engineering, West Pomeranian University of Technology in Szczecin, Piastów Ave. 50a, 70-311 Szczecin, Poland; daniel.sibera@zut.edu.pl; 3 Department of Chemical Organic Technology and Polymeric Materials, Faculty of Chemical Technology and Engineering, West Pomeranian University of Technology in Szczecin, Pułaskiego 10, 70-322 Szczecin, Poland; marcin.gano@zut.edu.pl

**Keywords:** photocatalytic CO_2_ reduction, nitrogen fixation, titanium dioxide, carbon spheres, microwave reactor

## Abstract

This paper presents new photocatalysts obtained by treating carbon spheres (CS) and TiO_2_ in a microwave reactor at a pressure of 20 atm and a temperature of up to 300 °C for 15 min and then depositing TiO_2_/CS composites on glass fibre cloths. Such highly CO_2_-adsorbing photocatalysts showed photoactivity in the simultaneous water-splitting process, generating H_2_, reducing CO_2_ to CO and CH_4_, and reducing N_2_ to NH_3_. In addition, calculations of the hydrogen balance involved in all reactions were performed. Adding 1 g of carbon spheres per 1 g of TiO_2_ maintained the high selectivity of nitrogen fixation at 95.87–99.5%, which was continuously removed from the gas phase into the water as NH_4_^+^ ions.

## 1. Introduction

The photocatalytic splitting of water into hydrogen necessary to reduce carbon dioxide into valuable hydrocarbons and reduce nitrogen into ammonia is one of the most significant research challenges [1,2,3] and is mainly performed by carrying out photosynthesis in a single reactor containing water, carbon dioxide, and nitrogen gas. In a triple system consisting of gaseous N_2_, CO_2_, and H_2_O (vapour), many of the following reactions are possible:

- A two-electron reaction of water splitting [4,5] resulting in the production of hydrogen:

H_2_O + 2h^+^ → ½O_2_ + 2H^+^ (1)

2H^+^ + 2e^−^ → 2H_2_ (2)

- A two-electron reduction of CO_2_ to carbon monoxide, following the equation below [6,7]:

CO_2_ + 2H^+^ + 2e^−^ → CO + H_2_O (3)

- A two-electron reaction towards formic acid production, proposed by Wang [3]:

CO_2_ + 2H+ + 2e^−^ → HCOOH (4)

- A four-electron reaction of the formation of formaldehyde:

CO_2_ + 4H^+^ + 4e^−^ → HCHO + H_2_O (5)

- Production of methane, which requires eight electrons [6,7]:

CO_2_ + 8H^+^ + 8e^−^ → CH_4_ + 2H_2_O (6)

- Nitrogen reduction, which follows the general reaction below [8]:

2N_2_ + 6H_2_O → 4NH_3_ + 3O_2_ (7)

From a practical point of view, to avoid troublesome and costly separation of products, selecting such conditions aims to obtain the highest possible amount and selectivity of the reaction to the desired product.

The kinds of products obtained in photocatalytic carbon dioxide reduction reactions depend on many parameters, primarily crystallite size, specific surface areas, surface zeta potentials, modifying additives, and type of photocatalyst, as well as others, such as temperature, pressure, reaction mixture composition, and phase composition. Liu et al. [9] showed that the proportion of desorption and photocatalytic conversion of CO_2_ to CO could be variable when higher process temperatures were used (100–200 °C). Particle dispersion and crystallinity of TiO_2_ on a hybrid adsorbent/photocatalyst supported on MgAl layered double oxides—MgAl(LDO)/TiO_2_—are also crucial in this case. Liu et al. [10] prepared TiO_2_–graphene nanocomposites via a chemical method from graphene oxide (GO) and TiO_2_ nanoparticles. The results showed that it was possible to photocatalytically reduce carbon dioxide to methanol (CH_3_OH) and methane (CH_4_) with efficiencies of 2.2 and 2.1 μmol/g/h, respectively, because of the synergistic effect between graphene and TiO_2_. Materials demonstrating a synergistic effect on methane production in the CO_2_ photoreduction process have also been developed. They contained not only TiO_2_ or graphene but also Pt or Au and were characterized by excellent activity [11,12].

The selectivity of the photocatalytic reduction of carbon dioxide can also be adjusted by the type of support used to deposit the photocatalyst. One example may be the work of Do et al. [13], where TiO_2_ modified with Fe, Co, Ni, or Cu was deposited on mineral basalt fibre films. For Co-TiO_2_/basalt fibre films, only methane was obtained, with a yield of 158–360 μmol/g/L after 8 h, which was explained by the improvement of CO_2_ adsorption and the synergy effect between the TiO_2_/basalt carrier and the prevention of electron–hole charge recombination. Wang et al. [6] prepared carbon-doped TiO_2_ with numerous oxygen vacancies and Ti^3+^ presence through Al reduction that restrained the recombination of photogenerated carriers. The obtained materials produced 4.1 μmol/g/h of CH_4_ and 2.5 μmol/g/h of CO under solar light, and 0.53 μmol/g/h of CH_4_ and 0.63 μmol/g/h of CO under visible light. Another common support material for TiO_2_ is mesoporous silica. It is characterized by large surface areas and well-ordered pore structures, which results in improved catalytic efficiency [14,15].

In this work, the preparation of nanocomposites based on TiO_2_ and carbon spheres by combining their properties under the influence of microwaves. We analysed the gas and water phase in the photoreactor, which has not been studied in previous works either by us or by other researchers. The use of a combination of microwave and temperature to connect carbon spheres and TiO_2_ improves the properties of the created hybrid photocatalyst, namely, by increasing CO_2_ adsorption and selectivity, and can prevent recombination of the electron–hole pair by creating a Ti–O–C chain [16], which is additionally enhanced by silica cloth.

## 2. Materials and Methods

### 2.1. Materials and Reagents

The following reagent-grade substances were used in the preparation of the carbon spheres:

- Resorcinol, C_6_H_4_(OH)_2_ (Chempur, Piekary Śląskie, Poland);
- 96 wt.% ethyl alcohol, C_2_H_5_OH (P.P.H. STANLAB, Lublin, Poland);
- 25 wt.% ammonia water solution, NH_4_OH (P.P.H. STANLAB, Lublin, Poland);
- 37 wt.% formaldehyde, HCHO (Chempur, Piekary Śląskie, Poland).

The TiO_2_/carbon spheres composites were obtained from previously prepared carbon spheres and pure AEROXIDE^®^ TiO_2_ P25 (Evonik Industries AG, Essen, Germany). They were applied on a fibreglass cloth with a density of 40 g/m^2^, supplied by Fiberglass Fabrics, Poland.

### 2.2. Preparation of Carbon Spheres

A total of 2.4 g of resorcinol was placed in the backer and dissolved in an aqueous alcohol solution consisting of 240 cm^3^ of distilled water and 96 cm^3^ of 96 wt.% ethyl alcohol. When the resorcinol was dissolved entirely, a 25 wt.% ammonia water solution was added dropwise until the pH reached 8–9. Then, 3.6 cm^3^ of formaldehyde (37 wt.%) was added. The mixture was stirred under ambient conditions to facilitate a polycondensation reaction for 24 h. After that, the content of the beaker was placed in a Teflon vessel and transferred to a microwave reactor Mangum II (Ertec-Poland, Wrocław, Poland). The process was carried out under a pressure of 20 atm for 15 min. Finally, the obtained material was dried in a laboratory dryer (Pol-Eko Aparatura, Wodzisław Śląski, Poland) at 80 °C for 24 h. The sample was carbonised under an argon atmosphere in an electronically controlled high-temperature tube furnace (HST 12/400, Carbolite Gero, Sheffield, United Kingdom) to obtain mesoporous carbon spheres. About 0.5 g of carbon material was weighed and poured onto a quartz boat in the centre of the quartz tube. The argon flow was controlled by an electronic flow meter (Brooks Instrument, Hatfield, PA, USA). The reaction was carried out under polythermal conditions from 20 to 350 °C at a heating rate of 1 °C/min, followed by a holding time of 2 h, and from 350 to 700 °C at a heating rate of 1 °C/min, followed by a holding time of 2 h. After this time, the carbonised sample was thoroughly washed until it reached a pH close to 7. For this purpose, a filtration set and a vacuum pump (Aga Labor, Warszawa, Poland) were assembled. The carbonaceous material was transferred to filter paper and washed with distilled water, controlling the pH of the filtrate. Once the target pH was reached, the sample was transferred to a dish and dried in a laboratory dryer for 48 h at 80 °C. The obtained material was marked with the symbol CS (carbon spheres). The names of the obtained photocatalyst samples and their composition are presented in Table 1.

### 2.3. Photocatalyst Bed Preparation

A total of 100 cm^3^ of 96 wt.% ethyl alcohol was measured and poured into a 150 cm^3^ beaker. A set number of spheres was weighed on an analytical balance and transferred to the beaker with ethanol. Then, 1 g of P25 titanium dioxide was weighed. As the carbon dissipated in the ethanol, titanium dioxide was gradually added. The beaker was left on the magnetic stirrer for 24 h. After that time, the beaker’s contents were poured into a Teflon container and placed in a microwave reactor (RM—Magnum II, Ertec-Poland, Wrocław, Poland). The process was carried out under a pressure of 20 atm, at a temperature not exceeding 300 °C, for 15 min. Then, the contents were poured into a flask and evaporated under a fume hood for 24 h. After the ethanol had evaporated, the beaker was placed in an oven (Pol-Eko Aparatura, Wodzisław Śląski, Poland) and dried at 80 °C for 24 h. Finally, the product was transferred to an agate mortar and carefully ground. The sample was labelled with the weight ratio of carbon spheres to TiO_2_ and an indication that it was from a microwave reactor (RM). In order to apply the photocatalyst on the support, 4 strips of glass fibre with dimensions of 40 × 80 × 0.25 mm (with a density of 40 g/m^2^, supplied by Fiberglass Fabrics, Poland) were prepared and weighed. Then, 0.5 g of the composite was weighed and mixed with 7 cm^3^ of distilled water (for samples with a weight ratio of CS: TiO_2_ < 1:1) or 4 cm^3^ of ethanol 96% and 3 cm^3^ of distilled water (for samples with a weight ratio of CS: TiO_2_ ≥ 1:1). The mixture was applied to the strips and then dried in an oven at 110 °C for 30 min. After drying, the prepared sample was weighed to determine the weight of the deposited photocatalyst. Then, the photocatalytic bed prepared this way was mounted inside a quartz photoreactor [17].

### 2.4. Photoactivity Measurements

The experiments were carried out in a cylindrical quartz reactor with a working volume of 392 cm^3^. Its scheme has been presented elsewhere [17]. Four Actinic BL TL-E Philips lamps with a total power of 88 W were used, emitting UV-A radiation with a wavelength of 350–400 nm. The lamps were placed to form a ring outside the reactor. The reactor and the rest of the equipment were enclosed in a thermostatic chamber in order to exclude other light sources and ensure a stable process temperature of 20 °C. Then, 3 cm^3^ of distilled water was poured into the reactor, and the photocatalyst, previously applied to glass fibre (FF 45 VLIES 50; 40 g/m^2^), was subsequently placed inside the reactor. The interior of the reactor was flushed with pure CO_2_ (Messer, Chorzów, Poland) for 30 min. After this time, the system was tightly closed, and the lamps were turned on. During the purge and the subsequent process, the gas was mixed using a peristaltic pump with a flow rate of 1.6 dm^3^/h. The process was run for 6 h, and gas samples were analysed every 2 h. In addition to CO_2_ (95%), 5% of the air was also present in the reactor.

### 2.5. Gas-Phase Analysis

The gas-phase composition was analysed using an SRI 310C gas chromatograph (SRI Instruments, Torrance, CA, USA) equipped with a column with a molecular sieve with a mesh size of 5Ӓ and an HID (Helium Ionization Detector). The carrier gas was helium. The analyses were performed under isothermal conditions at a temperature of 60 °C. The gas flow through the column was 60 cm^3^/min, and the volume of the test gas was 1 cm^3^. The content of the component in the gas phase was calculated in successive measurements based on the calibration curve.

### 2.6. Liquid-Phase Analysis

After the photocatalytic process, water was collected to determine the pH value and the ammonia content (NH_4_^+^ ions). The sensitive and economical Nessler method, which is typically applied for this purpose [18], was performed using a UV-Vis spectrophotometer V-650 (JASCO International Co., Tokyo, Japan) for this analysis.

### 2.7. Investigation of Surface Morphology

The surface morphology of the samples was examined using a TESCAN Vega3 scanning electron microscope (TESCAN, Brno, Czech Republic). The parameters of the SEM analysis were: accelerating voltage of 10 kV, magnification of 33.3k and 66.6k. Samples for SEM tests were first sputtered with a thin layer of chromium with a thickness of 5 nm to protect the sample against electric charge.

### 2.8. Textural Parameters and CO_2_ Adsorption Capacity Analysis

The study of low-temperature nitrogen adsorption at −196 °C was used to determine the specific surface area (S_BET_) and total pore volume (TPV) for all obtained materials. Measurements were performed on a Quadrasorb evo^TM^ Gas Sorption automatic system (Quantachrome Instruments, Anton Paar Group AB, Graz, Austria). Before each measurement, the samples were dried for 48 h at 80 °C in a laboratory oven and then outgassed at 250 °C under 1 × 10^−5^ mbar vacuum for 12 h using a MasterPrep multi-zone flow/vacuum degasser from Quantachrome Instruments. The Brunauer–Emmett–Teller (BET) equation determined the specific surface area. The surface area was determined in the 0.05–0.3 relative pressure range. The total pore volume was calculated from the volume of adsorbed nitrogen at relative pressure *p* = 0.99 × p_0_, where p_0_ is the saturated vapour pressure of nitrogen at −196 °C.

To determine the CO_2_ adsorption isotherms at 25 °C, a Quantachrome Instruments volume Quadrasorb™ analyser was used in the pressure range between 0.01 and 0.98 bar. Prior to the adsorption measurements, the samples were dried for 48 h at 80 °C in a drying oven and then outgassed at 250 °C for 12 h under reduced pressure using a Masterprep multi-zone flox/vacuum degasser from Quantachrome Instruments.

The surface area and CO_2_ sorption capacity at 25 °C values were calculated from the composite components’ mass fractions (listed in Table 1) according to the formulas below (Equations (8) and (9), respectively):

(8) $$S_{P25+CS\;RM}^{calc.}=\frac{m_{P25}}{m_{P25}+m_{CS\;RM}}\cdot S_{BET(P25)}+\frac{m_{CS\;RM}}{m_{P25}+m_{CS\;RM}}\cdot S_{BET(CS\;RM)}$$

(9) $${{CO}_{2}cap.}_{P25+CS\;RM}^{calc.}=\frac{m_{P25}}{m_{P25}+m_{CS\;RM}}\cdot {CO}_{2}\;{cap.}_{P25}+\frac{m_{CS\;RM}}{m_{P25}+m_{CS\;RM}}\cdot {CO}_{2}\;{cap.}_{CS\;RM}$$

where:

- $S_{P25+CS\;RM}^{calc.}$—theoretical surface area calculated, including measured BET specific surface area (S_BET_) [m^2^/g] for TiO_2_ P25 ($S_{BET(P25)}$) and CS RM ($S_{BET(CS\;RM)}$) and composite components’ mass fractions;
- $\frac{m_{P25}}{m_{P25}+m_{CS\;RM}}$—the mass fraction of TiO_2_ P25 photocatalyst in the composite;
- $\frac{m_{CS\;RM}}{m_{P25}+m_{CS\;RM}}$—the mass fraction of CS RM in the composite;
- ${{CO}_{2}cap.}_{P25+CS\;RM\;}^{calc.}$—the theoretical CO_2_ sorption capacity calculated on the basis of the measured CO_2_ sorption capacity [mmol CO_2_/g] for TiO_2_ P25 (${CO}_{2}\;{cap.}_{P25}$) and CS RM (${CO}_{2}\;{cap.}_{CS\;RM}$), and the composite components’ mass fractions.

## 3. Results

### 3.1. Characterisation of Materials

**Figure 1:**
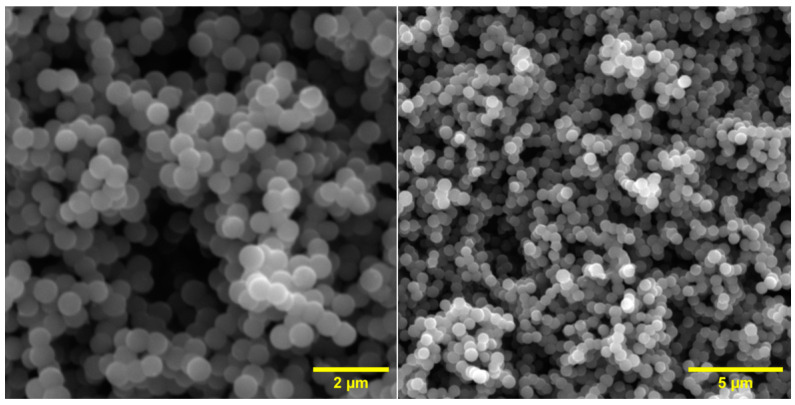
SEM images of unmodified carbon spheres.

SEM images of the pure carbon spheres are presented in Figure 1. It can be observed that the material is characterised by its smooth surface and spherical shape. Additionally, the surface morphology shows carbon spheres with a diameter of approximately 600–700 nm.

**Figure 2:**
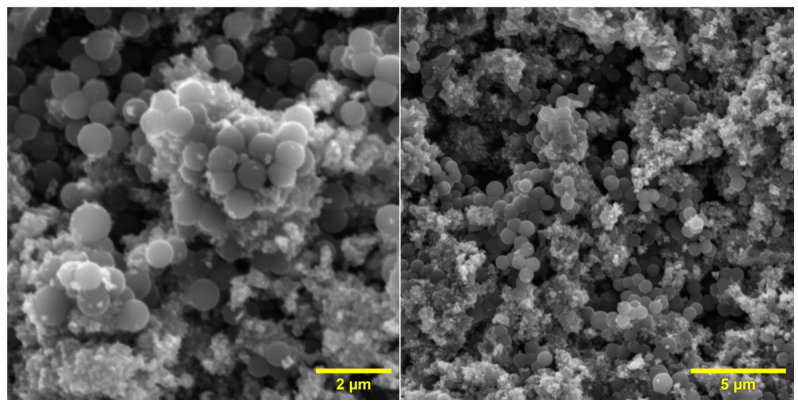
An example of the SEM surface morphology for the sample P25 + 0.5 CS RM.

The exemplary SEM photos for the sample P25 + 0.5 CS RM (Figure 2) show carbon spheres with diameters ranging from 600 to 1000 nm, along with TiO_2_ agglomerates. The carbon spheres are still characterised by their smooth surface and spherical shape. In the SEM pictures of other samples, not shown here, the only differences observed were in the number of carbon spheres, which were appropriate to their share of composites.

**Figure 3:**
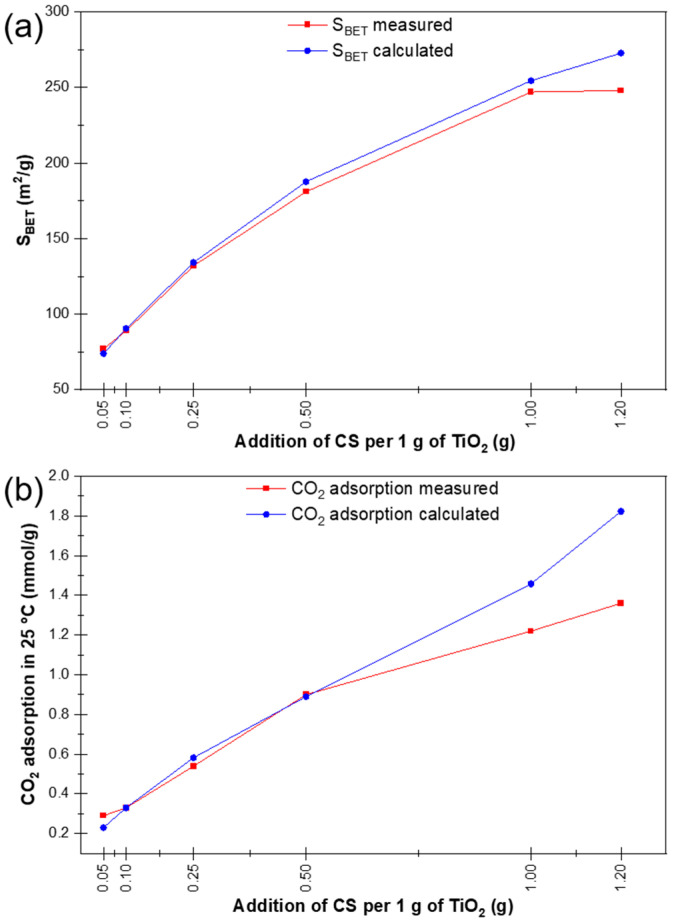
BET specific surface area (**a**) and CO_2_ adsorption at 25 °C (**b**), measured vs calculated from the mass fractions of the composite components of the studied samples.

Table 2 shows the measurements of the specific surface area (S_BET_) and CO_2_ adsorption at 25 °C for samples of photocatalysts with different mass ratios of TiO_2_ and carbon spheres [19]. It can be seen that both the specific surface area of the photocatalysts and the ability to adsorb CO_2_ increase with the amount of carbon spheres introduced into the P25. The table also presents the measured BET specific surface area compared with the specific surface area calculated from the mass fractions of the components. Similarly, the measured CO_2_ adsorption versus the adsorption calculated from the mass fractions of the individual components is compared. It can be seen that the total pore volume (TPV) increased to 0.33–0.43 cm^3^/g for all photocatalysts compared to the pure carbon spheres, which had a TPV of 0.26 cm^3^/g, with no apparent dependence on the amount of carbon spheres added. This increase in the total pore volume of the photocatalyst relative to the pure carbon spheres can be explained by the decrease in the number of micropores in favour of the formation of macropores as a result of the preparation of the photocatalyst at increased pressure (20 atm) and temperature (up to 300 °C) in the microwave reactor. The shape of the TiO_2_/CS agglomerates shown in Figure 2 suggests the creation of a new spatial structure. The data described above are shown in Figure 3.

Based on the relationship between the measured surface area and the calculated one (Figure 3a,b), it can be observed that with the addition of less than 1 g of carbon spheres, the measured values of specific surface area and CO_2_ sorption capacity are similar to the expected (calculated) values. However, the most significant changes between calculated and measured values—thus, a negative synergistic effect—can be seen by adding more than 1 g of carbon spheres. In this case, the measured values were lower than the calculated values.

### 3.2. Photoactivity Measurements

Table 3 presents the results of the measurements of the gas-phase composition (H_2_, CO, and CH_4_) and the content of ammonium NH_4_^+^ in the water phase for the tested samples. After 6 h, the hydrogen content increased from 0.35 μmol/g TiO_2_ for the P25 sample to 0.75 μmol/g TiO_2_ for the P25 + 0.05 CS RM sample, followed by 0.00 μmol/g TiO_2_ for the P25 + 1.2 CS RM sample. The CO content increased from 26.95 μmol/g TiO_2_ for pristine P25 to 31.64 μmol/g TiO_2_ for the P25 + 0.05 CS RM sample before gradually decreasing. In the case of methane, a systematic decrease in content to zero was observed for the P25 + 1.2 CS RM sample. The ammonia content in the aqueous phase increased from 586.56 μmol NH_4_^+^/g TiO_2_ for the P25 sample to 790 μmol NH_4_^+^/g TiO_2_ and 703 μmol NH_4_^+^/g TiO_2_ for the P25 + 0.05 CS RM and P25 + 0.25 CS RM samples respectively, before gradually decreasing to 143.59 μmol NH_4_^+^/g TiO_2_ (P25 + 1.2 CS RM).

**Figure 4:**
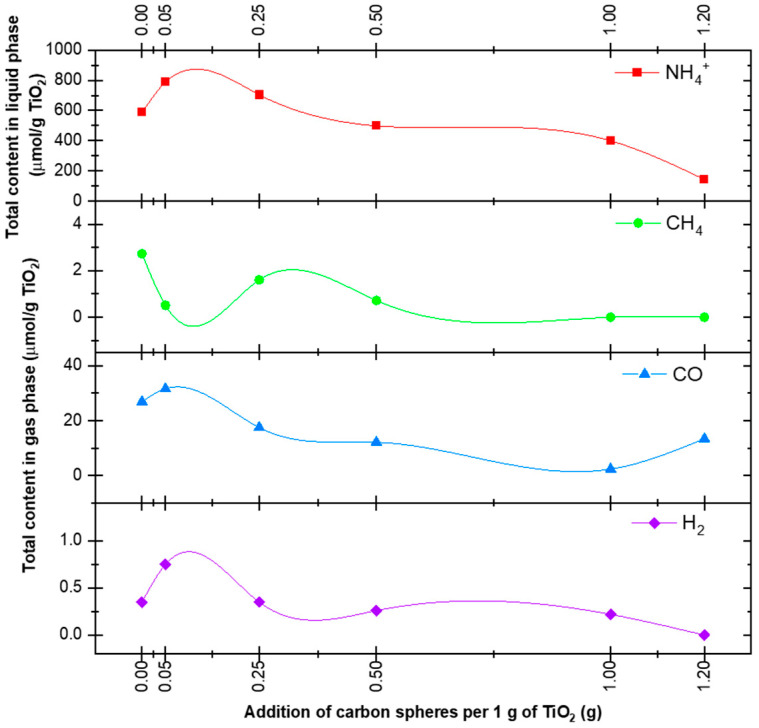
Products contained in the liquid phase (NH_4_^+^) and the gas phase (H_2_, CO, CH_4_) after processing for 6 h for different levels of carbon sphere addition per 1 g of TiO_2_.

The trends of the results presented in Table 3 are shown in Figure 4. As previously suggested, creating a new spatial structure with a greater proportion of macropores can be seen, especially in the range above 0.25–0.5 g CS/g TiO_2_, where the overall activity of the photocatalyst per 1 g of TiO_2_ decreases.

The hydrogen balance shown in Table 4 was determined by calculating the amount of hydrogen produced and the selectivity of the individual reaction products. The total amount of hydrogen was calculated, assuming that, apart from free H_2_, it will be used in the reduction reaction of carbon dioxide to carbon monoxide (10), in the reaction producing methane (11), and in the production of ammonia (12):

CO_2_ + H_2_ → CO + H_2_O (10)

CO_2_ + 4H_2_ → CH_4_ + 2H_2_O (11)

N_2_ + 3H_2_ → 2NH_3_ (12)

With these assumptions, the total amount of hydrogen was calculated, as shown in the last column of Table 4. Then, the selectivity of individual reactions was calculated using the hydrogen consumed.

The resulting selectivity to hydrogen from water splitting is very low, less than 0.06%, which is understandable. The hydrogen produced is immediately consumed, reducing CO_2_ and nitrogen. Additionally, the demanding eight-electron reduction of CO_2_ to methane proceeds with low selectivity, systematically decreasing from 1.19% for P25 to zero for the last two samples with the highest carbon sphere contents.

**Figure 5:**
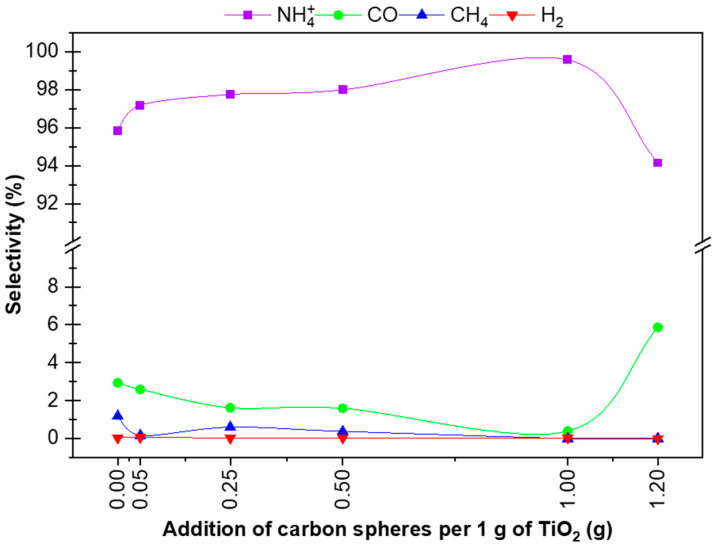
Calculated selectivities for hydrogen, carbon monoxide, methane and ammonia based on hydrogen balance for individual samples.

Based on the results presented in Figure 5, it can be concluded that there is competition between the reactions resulting in individual products, with the competition between the two-electron reduction of CO_2_ to CO and the three-electron reduction of nitrogen to ammonia (NH_4_^+^ ions) predominating. It can be seen that with higher carbon sphere contents when the selectivity to ammonia is somewhat lowered, the higher selectivity to CO ‘returns’. Additionally, the selectivity to ammonia remains high, from 95.84% for P25 to 99.57% for the P25 + 1 CS RM photocatalyst. Maintaining high levels of ammonium production and selectivity confirms the earlier suggestion [17] that the use of such a photocatalytic reactor design containing two phases of reactants and products, namely, the gas phase and water, causes the phenomenon of immediate absorption of the produced ammonia gas into the water upon the formation of the NH_4_^+^ ion, while the ammonia formation reaction moves towards the product in equilibrium. Additionally, the presence of HCO_3_^−^ and CO_3_^2−^ ions in CO_2_-saturated water accelerates this process.

**Figure 6:**
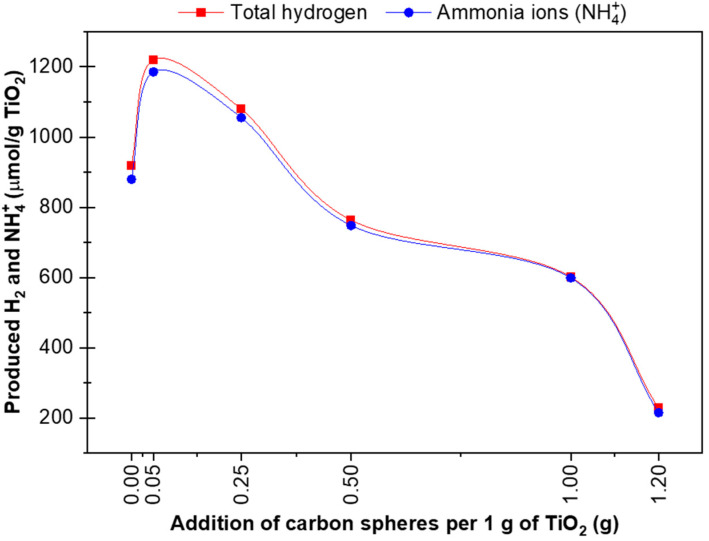
Total hydrogen produced vs hydrogen used in ammonia formation.

These results show that most of the hydrogen obtained from splitting water was used to produce ammonia, as shown in Figure 6, which shows the amount of hydrogen vs the amount of ammonia produced (NH_4_^+^ ions).

It should be emphasized that the simultaneous photoreduction of CO_2_ and the conversion of nitrogen to ammonia has not been widely described in the literature, which makes our research innovative. Since several factors affect the amounts of the product obtained (e.g., photocatalyst type, size, light source, temperature), it is difficult to objectively compare the results obtained here with those presented in other publications. However, an undoubted advantage of our system is the much lower lamp power (88 W) used. A number of publications have presented the results of CO_2_ reduction using high-power lamps, for example, Andrade et al. obtained 8 μmol·gTiO_2_^−1^·h^−1^ and 4 μmol·gTiO_2_^−1^·h^−1^ with the use of N-doped TiO_2_ and a 450 W Xe arc lamp [20]. Liu et al. produced 2.10 μmol·g^−1^·h^−1^ of CH_4_ and 2.20 μmol·g^−1^·h^−1^ of CH_3_OH using graphene-modified TiO_2_ under a lamp with a power of 250 W [10]. Experiments using Pt and Cu-modified TiO_2_ were presented in [21], where the products were: 4.9–66.0 μmol·g^−1^·h^−1^ of H_2_, 2.2–8.3 μmol·g^−1^·h^−1^ of CO, and 1.2–33.0 μmol·g^−1^·h^−1^ of CH_4_. A 200 W Xe lamp was used in this work. Similar results have been obtained using a 300 W lamp [14,22].

Additionally, previous reports concerning photocatalytic nitrogen fixation have shown that this process is mostly performed under high-power light sources. In experiments performed using modified TiO_2_ and water (similar to this publication), 38.4–419.0 μmol·g^−1^·h^−1^ of ammonia was obtained [23,24,25,26]. All of these processes were carried out using a 300 W Xe lamp.

The results presented in this publication prove that it is possible to simultaneously reduce CO_2_ and N_2_ fixation to ammonia under mild conditions, with high selectivity to NH_3_.

## 4. Conclusions

The microwave treatment of TiO_2_ and highly CO_2_-adsorbing carbon spheres under a pressure of 20 atm is a valuable method for forming photocatalysts, which, when mounted on a glass fibre fabric, will offer a higher simultaneous reduction of CO_2_ to H_2_, CO, and CH_4_ and N_2_ to ammonia compared to pure commercial TiO_2_ P25 under UV-A radiation. In order to calculate the selectivity of the photocatalyst towards the formation of hydrogen, carbon monoxide, methane, and ammonia, the balance of hydrogen involved in all of the mentioned reactions was calculated in both the gas phase and the water phase. Adding 0.25–0.5 g of carbon spheres per 1 g of TiO_2_ increased the total pore volume of the photocatalyst and its photoactivity during CO_2_ and nitrogen reduction. Within this content range of carbon spheres, a correlation was also observed between the specific surface area and the expected CO_2_ adsorption, both calculated and measured. For higher values of carbon sphere content, the hydrogen obtained from water splitting disappeared from the post-reaction gases because it was wholly consumed by the formation of the carbon monoxide and ammonia reactions, which compete with each other. In these ranges of carbon sphere content (1 g CS/1 g TiO_2_), the eight-electron methane formation reaction, which is the most difficult to conduct, is absent. At up to 1 g CS/1 g TiO_2_, the high selectivity of nitrogen fixation to ammonia was also obtained, with 95.87–99.5% values, due to the rapid absorption of ammonia formed in water to NH_4_^+^ ions.

## Figures and Tables

**Table 1:** Composition of prepared photocatalysts.

Sample Name	Amount of TiO_2_ (g)	Amount of Carbon Spheres (g)
P25	1.00	0.00
P25 + 0.05 CS RM	1.00	0.05
P25 + 0.1 CS RM	1.00	0.10
P25 + 0.25 CS RM	1.00	0.25
P25 + 0.5 CS RM	1.00	0.50
P25 + 1 CS RM	1.00	1.00
P25 + 1.2 CS RM	1.00	1.20

**Table 2:** Summary of the results for the low-temperature nitrogen adsorption and CO_2_ adsorption at 25 °C compared with calculated values for composites based on previously prepared carbon spheres and P25 obtained using a microwave reactor [19]. * Calculated from the mass fractions of composite components (Equations (8) and (9)).

Sample Name	Specific Surface Area	Calculated Surface Area *	Total Pore Volume	CO_2_ Sorption Capacity at 25 °C	Calculated CO_2_ Sorption Capacity at 25 °C *
	(m^2^/g)	(m^2^/g)	(cm^3^/g)	(mmol/g)	(mmol/g)
P25	54	-	0.40	0.12	-
CS	455	-	0.26	2.43	-
P25 + 0.05 CS RM	77	73	0.33	0.29	0.23
P25 + 0.1 CS RM	89	91	0.34	0.33	0.33
P25 + 0.25 CS RM	132	134	0.43	0.54	0.58
P25 + 0.5 CS RM	181	188	0.37	0.90	0.89
P25 + 1 CS RM	247	255	0.33	1.22	1.46
P25 + 1.2 CS RM	248	273	0.36	1.36	1.82

**Table 3:** The composition of the gas phase in the photoreactor and the content of ammonium ions in the liquid phase for the tested samples of photocatalysts with different carbon sphere contents.

Sample Name	Total Content in the Gas Phase after Processing for 6 h (μmol/g TiO_2_)			Total Content of NH_4_^+^ in the Liquid Phase after Processing for 6 h (μmol NH_4_^+^/g TiO_2_)
	H_2_	CO	CH_4_	
P25	0.35	26.95	2.73	586.56
P25 + 0.05 CS RM	0.75	31.64	0.51	790.17
P25 + 0.25 CS RM	0.35	17.48	1.61	703.69
P25 + 0.5 CS RM	0.26	12.17	0.72	498.77
P25 + 1 CS RM	0.22	2.37	0.00	399.41
P25 + 1.2 CS RM	0.00	13.41	0.00	143.59

**Table 4:** Hydrogen balance in the products of the gas and liquid phases in the photoreactor for the tested samples of photocatalysts with different carbon sphere contents.

Sample Name	H_2_ *		CO **		CH_4_ ***		NH_4_^+^ ****		ΣH_2_ (μmol)
		Selectivity (%)		Selectivity (%)		Selectivity (%)		Selectivity (%)	
	(μmol)		(μmol)		(μmol)		(μmol)		
P25	0.35	0.04	26.95	2.94	10.92	1.19	879.84	95.84	918.06
P25 + 0.05 CS RM	0.75	0.06	31.64	2.59	2.04	0.17	1185.26	97.18	1219.69
P25 + 0.25 CS RM	0.35	0.03	17.48	1.62	6.44	0.60	1055.54	97.75	1079.81
P25 + 0.5 CS RM	0.26	0.03	12.17	1.59	2.88	0.38	748.16	97.99	763.47
P25 + 1 CS RM	0.22	0.04	2.37	0.39	0.00	0.00	599.12	99.57	601.71
P25 + 1.2 CS RM	0.00	0.00	13.41	5.86	0.00	0.00	215.39	94.14	228.80

* H_2_ in the gas phase; ** H_2_ used in Reaction (10); *** H_2_ used in Reaction (11); **** H_2_ used in Reaction (12).

## Data Availability

Not applicable.
